# Anticoagulants versus Left Atrial Appendage Occlusion in Patients with Atrial Fibrillation and Co-Morbid Thrombocytopenia

**DOI:** 10.3390/jcm12247637

**Published:** 2023-12-12

**Authors:** Wiktoria Kowalska, Katarzyna Mitręga, Anna Olma, Tomasz Podolecki, Zbigniew Kalarus, Witold Streb

**Affiliations:** 1 Doctoral School, Division of Medical Sciences in Zabrze, Medical University of Silesia, 40-055 Katowice, Poland; 2 Department of Cardiology and Angiology, Silesian Center for Heart Diseases, 41-800 Zabrze, Poland; 3 Department of Cardiology and Electrotherapy, Faculty of Medical Sciences in Zabrze, Medical University of Silesia, 40-055 Katowice, Poland

**Keywords:** atrial fibrillation, left atrial appendage occlusion, thrombocytopenia

## Abstract

Left atrial appendage closure (LAAC) is an alternative approach to anticoagulants. Nonetheless, data regarding the outcomes of LAAC procedures in patients with thrombocytopenia remain lacking. The primary objective was to determine the incidence of the composite endpoint comprising ischemic stroke, intracranial hemorrhage, major bleeding, and cardiac cause of death among patients with atrial fibrillation (AF) and thrombocytopenia who were either undergoing LAAC or receiving oral anticoagulants. The secondary endpoint was the determination of total mortality. Data from a prospective, single-center registry of patients undergoing LAAC procedures were analyzed. A subset of 50 consecutive patients with thrombocytopenia were selected. Thrombocytopenia was defined as a thrombocyte count below 150,000. Subsequently, from patients hospitalized with AF receiving oral anticoagulants, 50 patients were further chosen based on propensity score matching, ensuring comparability with the study group. The primary endpoint occurred in 2% of patients in the LAAC group and 10% of patients in the non-LAAC group (*p* = 0.097). Additionally, a significant difference was noted in the occurrence of the secondary endpoint, which was observed in 0% of patients in the LAAC group and 10% of patients in the non-LAAC group (*p* = 0.025). In patients with thrombocytopenia the LAAC procedure improves prognosis compared with continued anticoagulant treatment.

## 1. Introduction

Advances in pharmacotherapy and an increased availability of direct oral anticoagulants (DOACs) have significantly enhanced the safety of preventing thromboembolic events in patients with atrial fibrillation (AF). However, adverse events associated with the use of both DOACs and vitamin K antagonists (VKAs) continue to be reported. Pharmacological treatment in patients with thrombocytopenia who require thromboembolic prophylaxis is exceptionally challenging due to the higher risk of bleeding and higher mortality in these patients [1].

Since many large trials testing the safety of DOACs excluded patients with thrombocytopenia, there are no official treatment guidelines for these patients [2,3,4,5]. It has been shown that in comparison to warfarin, DOACs are linked to a lower risk of major bleeding (4.10 vs. 8.26 per person years, *p* < 0.001) and a lower risk of ischemic stroke and systemic embolism in patients with thrombocytopenia (3.16 vs. 3.94 per person years, *p* < 0.001) [6], which suggests that oral anticoagulation with DOACs should be preferred in this population. Regardless of these favorable results, VKAs are still widely used for ischemic stroke prevention in patients with AF due to economic reasons.

However, several authors described cases of thrombocytopenia observed after DOACs [7,8,9]. This complication has been observed in patients treated with rivaroxaban and apixaban. Moreover, thrombocytopenia is mentioned in apixaban’s manufacturer’s possible side effects list.

It is also worth mentioning that thrombocytopenia itself is a predictor of bleeding included in the HEMORR2HAGES scale that allows risk stratification on presentation with intracranial hemorrhage [10].

On the other hand, it is crucial to remember that the presence of thrombocytopenia does not reduce the risk of thromboembolism. Some studies show that patients with immune thrombocytopenia are at increased risk of venous thromboembolism [11,12].

Several studies have proven that percutaneous left atrial appendage closure (LAAC) is a safe alternative to oral anticoagulants, reducing bleeding risk and mortality [13,14,15,16]. Until now, only one study has analyzed the safety and efficacy of LAAC in patients with thrombocytopenia [17]. Zhang et al. compared the results of LAAC in patients with and without chronic thrombocytopenia, defined as a platelet count below 100,000/µL. Their results suggest that the LAAC procedure can be safe in patients with thrombocytopenia; however, patients with this condition should be carefully monitored for bleeding, since thrombocytopenia was an independent risk factor for any bleeding [17].

The primary objective of this study was to determine the incidence of the composite endpoint comprising ischemic stroke, hemorrhagic stroke major bleeding, and cardiac cause of death among patients with atrial fibrillation and thrombocytopenia who were either undergoing LAAC or receiving oral anticoagulants. The secondary endpoint was the determination of total mortality in both groups.

## 2. Materials and Methods

### 2.1. Study Population

Data from a prospective, single-center registry of patients undergoing LAAC procedures between 2011 and 2021 were analyzed. From this dataset, a subset of 50 consecutive patients with thrombocytopenia were selected. Thrombocytopenia was defined as a thrombocyte count below 150,000/µL (LAAC group).

Additionally, data from 3200 patients hospitalized for various reasons with AF who were using oral anticoagulants for cardiovascular stroke prevention were analyzed retrospectively. From this population, 150 patients with concomitant thrombocytopenia were selected. Among them, 50 patients (non-LAAC group) were further chosen based on propensity score matching, ensuring comparability in terms of HAS-BLED, type of AF, ischemic stroke, and history of major bleeding with the study group.

**Figure 1:**
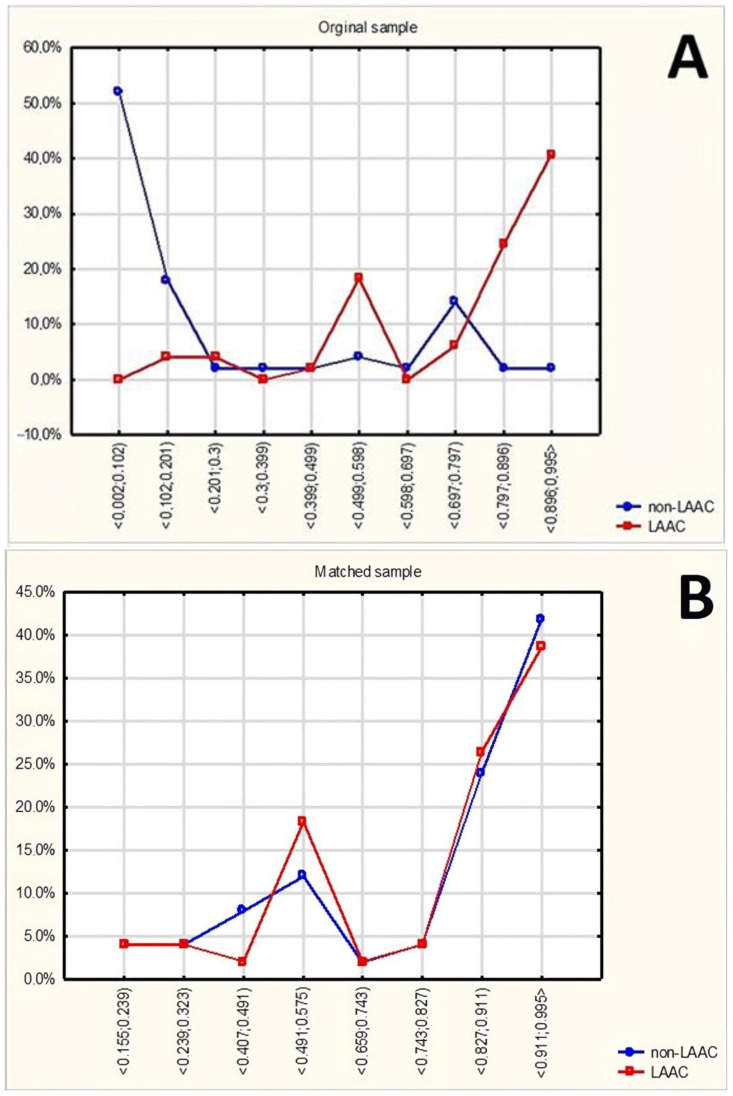
Distribution of propensity scores in the original sample (**A**) and after the matching procedure (**B**).

The characteristics of both groups before matching are given in Table 1. Figure 1 shows the distribution of propensity scores in the original sample and after the matching procedure, reflecting the quality of the resulting match. The characteristics of the matched groups are given in Table 2.

The etiology of thrombocytopenia was not recorded in this study.

### 2.2. Indications for Left Atrial Appendage Closure (LAAC)

Patients qualified for the LAAC procedure were patients with high thromboembolic risk (preferably CHA2DS2-VASc ≥ 2 for men or ≥3 for women). Patients undergoing the LAAC procedure after the release of 2020 ESC guidelines for the diagnosis and management of atrial fibrillation were qualified in accordance with them—LAA occlusion may be considered for stroke prevention in patients with AF and contraindications for long-term anticoagulant treatment (e.g., intracranial bleeding without a reversible cause) (Class IIB, level B). Previously, 2016 ESC guidelines for AF management were used (Class IIB, level B) [18].

### 2.3. Left Atrial Appendage Closure (LAAC)

LAAC procedures were conducted using three types of occluders—Amplatzer™ Amulet™ (St. Jude Medical, Minneapolis, MN, USA) (*n* = 39, 78%), Amplatzer Cardiac Plug (ACP, AGA, St. Jude Medical, Minneapolis, MN, USA) (*n* = 8, 16%), or Watchmen (Boston Scientific, Marlborough, MA, USA) (*n* = 3, 6%). The techniques of implantation differed from one device to the next and were performed according to the manufacturer’s instructions. A general technique of the procedure is presented below.

LAA morphology and precise measurements were taken the day before the procedure and then repeated at the beginning to ensure the correct device selection. An antibiotic prophylactic was used in each patient before the procedure (2 g of cefazolin intravenously).

The procedures were performed under general anesthesia or deep sedation, under the guidance of transesophageal echocardiography. Venous access was obtained via the right femoral vein, and a guidewire and vessel dilator were inserted into the vein. The interatrial septum was punctured using a standard trans-septal access system, under the guidance of transesophageal echocardiography. A bolus of unfractioned heparin was administered to obtain an activated clotting time (ACT) over 250 ms along with the puncture.

Afterward, a sheath was advanced into the left upper pulmonary vein and redirected into the left atrial appendage (LAA) over a pigtail catheter.

Then, the occluder was advanced in a delivery sheath and deployed in the LAA; the position was confirmed via fluoroscopy and transesophageal echocardiography. After performing a tug test, the device was finally released.

Criteria for optimal deployment were used as advised by the manufacturer (e.g., PASS for Watchmen, CLOSE for Amulet).

A control transesophageal echocardiography at the end of the procedure confirmed the correct position of the device and the presence of peri-device leaks.

### 2.4. Antiplatelet Therapy following the Procedure

If no contraindications were present, patients, after LAAC, were discharged on dual antiplatelet therapy (aspirin 75 mg daily plus clopidogrel 75 mg daily), which continued for 2–3 months. Decisions about discharging patients only on one antiplatelet drug (aspirin—10.9% or clopidogrel—7.3%) were made individually depending on individual morphology results (e.g., thrombo- or pancytopenia) and after a careful analysis of individual bleeding risk. If the control transesophageal echocardiography showed no signs of thrombus or leak after three months, the patients were left on aspirin only (67.3%).

Detailed information about the antiplatelet therapy and anticoagulants are presented in Table 3.

### 2.5. Follow-Up

In the 3rd month from discharge, patients from the LAAC group attended a clinical visit, where transesophageal echocardiography was performed, and details about current medications and clinical events were noted. Patients from the control group were contacted via phone and enquired about their current medications and clinical events.

At the 12th month, both groups were contacted via phone and enquired about current medications, significant bleeding, stroke (hemorrhagic or ischemic), and myocardial infarction. No patients were lost to follow-up.

### 2.6. Clinical Outcomes

The primary composite endpoint encompassed ischemic stroke, intracranial hemorrhage, major bleeding, and cardiac-related deaths in the patient population. The secondary endpoint was overall mortality. Major bleeding was defined as bleeding necessitating hospitalization due to a life-threatening situation. The efficacy of the LAAC procedure was assessed by comparing the yearly anticipated risks of stroke and bleeding derived from the CHA2DS2-VASc and HAS-BLED scales with the observed rates of stroke and significant bleeding.

### 2.7. Statistical Analysis

Original samples were compared using U-Mann–Whitney or chi-square. Propensity score matching was used to correct differences between the two groups resulting from non-random selection of the LAAC and non-LAAC groups. This consisted of matching each case from the LAAC group with a corresponding case from the non-LAAC group so that the distribution of analyzed characteristics was as similar as possible in both groups. As multiple criteria were considered, logistic regression was used to calculate the propensity score. The nearest-neighbor method was used to associate cases via propensity score matching. Further calculations were carried out on the groups obtained after matching.

Survival was analyzed with a long-rank test and presented on a Kaplan–Meier plot. A *p*-value of less than 0.05 was considered statistically significant. Statistical analysis was performed using Statistica version 13 software.

## 3. Results

No patients were lost to follow-up at 12 months. The LAAC procedure was successful in all patients; no periprocedural complications were observed in the LAAC group.

**Figure 2:**
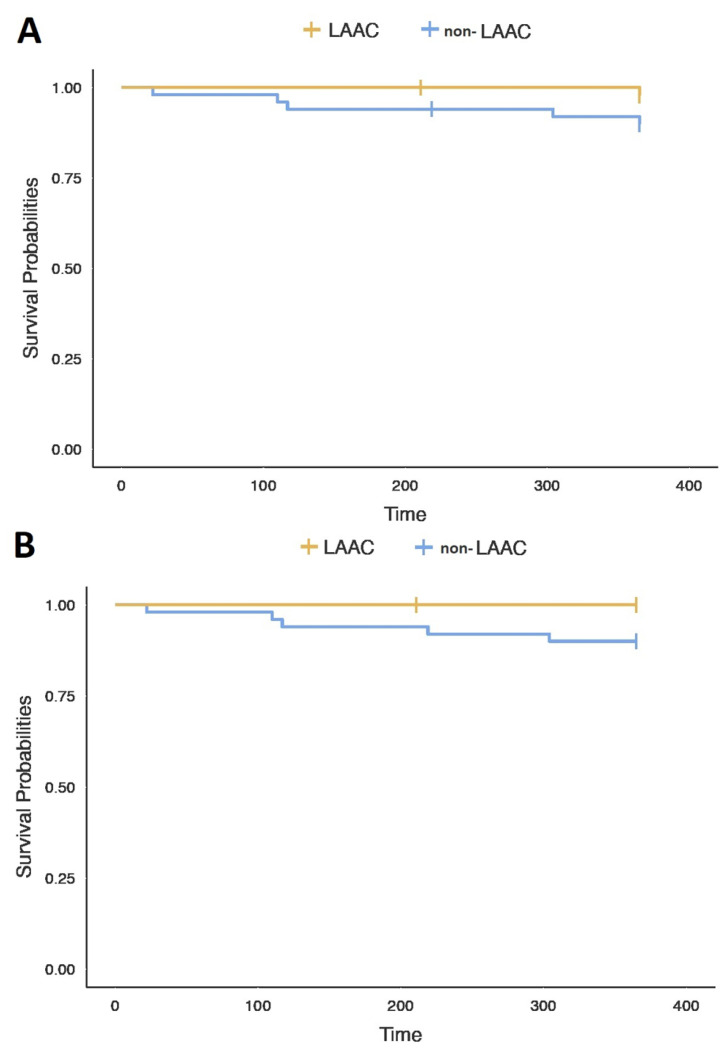
Event-free survival curves for primary (**A**) and secondary endpoint (**B**).

During the 12-month follow-up, ischemic stroke occurred in one (2%) patient in the LAAC group and in one (2%) patient in the non-LAAC group (*p* = 0.99). Only two significant bleedings were observed in the non-LAAC group, and included a cardiac tamponade and bleeding from the gastrointestinal tract, both requiring urgent hospitalization, while no major bleeding was observed in the LAAC group (*n* = 2, 4% vs. *n* = 0, 0%, *p* = 0.14). The incidence of a primary composite endpoint was higher in the non-LAAC group than in the LAAC group, but it was statistically insignificant (*n* = 5, 10% vs. *n* = 1, 2%, *p* = 0.09). However, there was a significant difference in the occurrence of the secondary endpoint, which was observed in 0 (0%) in the LAAC group and 5 (10%) in the non-LAAC group (*p* = 0.03). Detailed results are presented in Table 4. Event-free survival curves are presented in Figure 2.

The predicted median risks of thromboembolic events calculated from the CHA_2_DS_2_-VASc score were, respectively, 6.7% vs. 4.0% for the LAAC and non-LAAC groups (*p* = 0.55). The reduction in thromboembolic events in the LAAC group was 70.15%. Also, the stroke rate observed in the non-LAAC group was 50% lower than expected. The mean difference of 2.7% favored the LAAC procedure (*p* = 0.023).

The bleeding risk, calculated using the HAS-BLED score, was higher in the LAAC group compared to the non-LAAC group (2.96 ± 2.24 vs. 2.44 ± 2.44), with a mean difference of 0.85% (*p* < 0.01). Interestingly, the observed bleeding rate in the non-LAAC group exceeded the expected rate by 43.26%. In contrast, the LAAC group exhibited a bleeding rate that was 100% lower than expected. This translated to a mean difference of 2.7% (*p* = 0.02) between the observed and expected bleeding rates in the LAAC group.

The control transesophageal echocardiography showed two incidents of device-related thrombus (DRT) and two incidents of peri-device leak (PDL) ≤ 5 mm. None of the patients with a DRT detected suffered from ischemic stroke in the further observation. However, one of the patients that was diagnosed with peri-device leak experienced an ischemic stroke during the 12-month follow-up.

## 4. Discussion

To the best of our knowledge, this study is the first to compare the outcomes of the LAAC procedure with oral anticoagulant therapy in patients suffering from AF and concurrent thrombocytopenia. Our research demonstrates that LAAC is a secure method for preventing strokes in patients with thrombocytopenia. The major findings of the study are that the study reported a 100% success rate for the LAAC procedure, with no peri-procedural complications observed, and thus the LAAC procedure can be assumed to be safe and feasible to study in patients with concurrent thrombocytopenia; choosing LAAC over oral anticoagulation in patients with thrombocytopenia results in reduced major bleeding events; and patients with thrombocytopenia undergoing LAAC have a better prognosis in terms of overall mortality compared to patients treated with oral anticoagulants.

The results of one of the largest trials, PROTECT-AF, proved LAAC to be non-inferior to warfarin in terms of prevention of stroke, systemic embolism, and cardiovascular death [13]; however, patients undergoing the procedure were exposed to procedure-related safety events like cardiac tamponade and peri-procedural stroke. Another multicenter, randomized study, PRAGUE-17, compared LAAC with direct oral anticoagulant (DOAC) therapy, showing a non-inferiority of the procedure. At the median of almost 20 months of follow-up, there was no difference between groups in composite endpoints—stroke/TIA, clinically significant bleeding, and cardiovascular death [14]. So far, only one study has analyzed the outcomes of LAAC in patients with thrombocytopenia by comparing patients undergoing the procedure with thrombocytopenia and with normal platelet counts [17]. Zhang et al. reported a reduction in clinical thromboembolism by 100% and bleeding by 42.47% in patients with thrombocytopenia undergoing LAAC, but low platelet count was an independent predictor of any bleeding events. This study reported a higher incidence of minor bleeding; however, the difference in the occurrence of major bleeding did not reach statistical significance.

As mentioned earlier, it is important to note that patients with thrombocytopenia do not have a reduced risk of thromboembolic events [11,12]. Even though the CHA2DS2-VASc score was not different between the groups, patients undergoing LAAC had a higher incidence of ischemic stroke in their medical history (22 vs. 6%, 0.04). Most of these patients suffered from ischemic strokes and bleeding episodes, indicating a significantly elevated risk for both thromboembolic events and bleeding complications. Considering the information provided, it can be inferred that the disparities in baseline characteristics between patients in the LAAC and non-LAAC groups were likely due to the presence of specific indications for the LAAC procedure. For instance, one patient experienced an ischemic stroke while on DOAC therapy, and another necessitated long-term dual-antiplatelet therapy following acute coronary syndrome. These situations align with the established indications for the LAAC procedure, shedding light on the divergent baseline features observed in the LAAC group patients.

During the 12-month follow-up, the ischemic stroke incidence after matching was the same for both groups (2 vs. 2%, *p* = 0.99). It is worth noting that while a considerable number of studies have demonstrated a significant reduction in stroke risk following LAAC, some studies indicate that the incidence of ischemic stroke after LAAC is comparable to the one observed in patients treated with direct oral anticoagulants [19,20,21]. Some recent data suggest that LAAC is most beneficial in patients with a high bleeding risk and a sufficiently low ischemic stroke risk, which should perhaps be considered in the decision-making process [22,23]. Another clinical issue that Galloo et al. highlighted is recurrent strokes despite adequate anticoagulant therapy. Their study employed LAAC as an adjunctive therapy alongside oral anticoagulants in patients experiencing recurrent stroke after exclusion of other plausible causes, demonstrating high feasibility and safety [24].

Furthermore, the occluder itself can be a source of thrombotic material. A recent study analyzed the predictors of device-related thrombus (DRT) following the LAAC procedure. It is worth drawing attention to a relatively high occurrence of DRT—at their last known follow-up, over 25% of patients had DRT. In this study, DRT was associated with a higher risk of the composite endpoint of death, ischemic stroke, or systemic embolization. In the multivariable analysis, five risk factors were identified—hypercoagulability disorder, pericardial effusion, renal insufficiency, implantation depth > 10 mm from the pulmonary vein limbus, and non-paroxysmal atrial fibrillation. Interestingly, medication at discharge did not have an impact on DRT [25].

In our study the device-related thrombus was identified in two of our patients. In the first case, the patient was discharged on single-antiplatelet therapy (aspirin) due to a low platelet count (65,000 platelets per microliter at discharge), and 80 mg of enoxaparin once daily was added. In the second case, the patient continued standard dual-antiplatelet therapy, which suggests that it might not be sufficient in some patients. After 3 and 2 months, respectively, the thrombus resolved in both cases. Importantly, none of the patients with device-related thrombus detected during the 3rd-month visit experienced an ischemic stroke during the 12-month follow-up period.

Despite the fact that in the initial analysis of PROTECT-AF [13] small peri-device leaks (PDLs) were not associated with ischemic stroke or systemic embolism, a later study proved that a PDL ≤ 5 mm was in fact associated with a higher risk of thromboembolism at 1 year from the procedure [26]. Interestingly, a PDL ≤ 5 mm at the 45th day after the Watchman LAAC procedure was not associated with an increased risk of ischemic stroke. Hence, it can be concluded that an early recognition of PDL and a prompt reaction could prevent a future ischemic stroke. One of the patients of our study, after LAAC with the Watchman device, had a PDL ≤ 5 mm observed at the control echocardiography and experienced an ischemic stroke in the further observation period.

The difference in the baseline HAS-BLED scores within this study population present before matching primarily stems from pre-existing bleeding events during anticoagulation therapy, which served as an indication for the LAAC procedure. In this study, two-thirds (66%) of patients eligible for LAAC had experienced previous bleeding episodes. Current research indicates that LAAC significantly reduces bleeding risk by 55–62.2% [21,25,27], a trend consistent with our study findings—we observed a remarkable 100% reduction in bleeding events among patients in the LAAO group.

Pastori et al. showed a more than two-fold mortality rate rise in patients with AF and moderate–severe thrombocytopenia (3.8%/year vs. 9.9%/year, *p* = 0.01) [11]. Multiple authors have suggested that thrombocytopenia is a risk factor for higher mortality in the presence of cardiovascular comorbidities like heart failure with reduced ejection fraction or coronary heart disease. The mortality was inversely proportional to the platelet count and varied between 15% and 30% [28,29,30]. The mortality rate in this study was not far from the mortality rates described in the literature [29,30,31]. Despite a higher preliminary burden of concomitant diseases in the LAAC group, we observed a significant difference in the overall mortality between the groups—0% in the LAAC group and five (10%) in the non-LAAC group (*p* = 0.03).

The guidelines for AF management of the European Society of Cardiology [18] clearly state that antithrombotic therapy has never been analyzed in a randomized trial and it is mainly based on historical studies. Antiplatelet therapy has been studied most profoundly in patients with thrombocytopenia and acute coronary syndrome, and data on the use of these drugs in patients with thrombocytopenia and different indications are scarce. When choosing a method of stroke prevention in patients with co-morbid thrombocytopenia, it must be kept in mind that a potentially life-long antiplatelet therapy follows the LAAC procedure and it is an inevitable part of the treatment.

## 5. Conclusions

The results of this study suggest that the LAAC procedure and postprocedural therapy employed could be considered safe for patients with AF and thrombocytopenia. For individuals with thrombocytopenia requiring cerebral stroke prophylaxis due to atrial fibrillation, the LAAC procedure has been shown to improve prognosis compared to continuing anticoagulant treatment. Further clinical trials are necessary to evaluate the potential benefits of LAAC in patients with co-morbid thrombocytopenia.

### Study Limitations

The small size of the patient group and the differences in baseline characteristics may have affected the results and made them excessively optimistic in terms of the LAAO procedure’s results. On the other hand, the relatively short follow-up time of 12 months might have not been enough to demonstrate the full benefit of the LAAO procedure.

While hospitalizations in the LAAO group were planned, some of the hospitalizations in the non-LAAO group were urgent (e.g., chronic-heart-failure-related, acute coronary syndrome), resulting in differences in baseline characteristics—the incidence of CHF and the intake of antiplatelet drugs.

The antiplatelet regimen following the LAAC procedure was individualized for each patient according to their morphology findings, which did not provide one repeatable scheme. However, the alterations in the treatment seemed inevitable.

## Figures and Tables

**Table 1:** Study group characteristics before propensity score matching.

	Non-LAAC	LAAC	*p*
	*N* (%)	*N* (%)	
Male sex	35(70%)	35 (70%)	1.00
Paroxysmal AF	31 (62%)	5 (12%)	<0.01
Persistent AF	5 (10%)	2 (4%)	0.39
Permanent AF	14 (28%)	42 (84%)	<0.01
CHF	32 (64%)	14 (28%)	<0.01
HA	35 (70%)	41 (82%)	0.24
DM	13 (26%)	12 (24%)	1.00
Past intracranial hemorrhage	2 (4%)	5 (10%)	0.44
Ischemic stroke	3 (6%)	11 (22%)	0.04
CKD	20 (40.8%)	17 (34%)	0.54
History of major bleeding	17 (39.5%)	33 (66%)	0.01
	Mean ± SD	Mean ± SD	
Age (years)	72l.28 ± 8.91	74.72 ± 6.80	0.06
LVEF [%]	43.84 ± 14.83	47.14 ± 12.19	0.29
CHA2DS2-VASc	4.08 ± 1.718	4.02 ± 1.169	0.86
HAS-BLED	1.82 ± 1.219	3.10 ± 0.814	<0.01
RBCs [10^6^/µL]	4.29 ± 0.521	4.44 ± 0.605	0.19
HGB [mmol/L]	8.59 ± 1.610	8.27 ± 1.050	0.81
HCT [%]	38.84 ± 4.713	39.89 ± 4.702	0.22
PLTs [10^3^/µL]	127.36 ± 21.826	119.56 ± 28.332	0.09
Creatinine [µmol/L]	113.23 ± 66.297	114.04 ± 66.620	0.82

Abbreviations: AF—atrial fibrillation; CHF—chronic heart failure; CKD—chronic kidney disease; DM—diabetes mellitus; HA—arterial hypertension; HCT—hematocrit; HGB—hemoglobin; LVEF—left ventricular ejection fraction; PLTs—platelets; RBCs—red blood cells.

**Table 2:** Study group characteristics after propensity score matching.

	Non-LAAC	Variance	LAAC	Variance
Age (years)	73.28	79.43	82.32	51.04
LVEF (%)	43.84	219.97	44.68	28.02
RBCs [10^6^/µL]	4.29	0.27	3.94	0.14
HGB	8.59	2.59	6.9	0.77
HCT [%]	38.84	22.22	34.43	12
PLTs [10^3^/µL]	127.36	476.24	133.76	785.69
Creatinine [µmol/L]	113.23	4395.33	135.26	786.4

Abbreviations: HCT—hematocrit; HGB—hemoglobin; LVEF—left ventricular ejection fraction; PLTs—platelets; RBCs—red blood cells.

**Table 3:** Antiplatelet therapy and anticoagulation administered at discharge.

	Non-LAAC	LAAC	*p*
ASA + Clopidogrel—*n* (%)	7 (14)	40 (80)	<0.001
ASA only—*n* (%)	2 (4)	3 (6)	0.60
Clopidogrel only—*n* (%)	1 (2)	3 (6)	0.29
DOAC—*n* (%)	30 (6)	1 (2)	<0.001
VKA—*n* (%)	13 (26)	0 (0)	<0.001
Heparin—*n* (%)	2 (4)	1 (2)	0.46

Abbreviations: ASA—acetylsalicylic acid; DOAC—direct oral anticoagulant; VKA—vitamin K antagonist.

**Table 4:** Results after 12-month observation.

	Non-LAAC (*n* = 50)	LAAC Group (*n* = 50)	*p*-Value
Ischemic stroke—*n* (%)	1 (2)	1 (2)	0.99
Intracranial hemorrhage—*n* (5%)	0 (0)	0 (0)	0.99
Major bleeding—*n* (%)	2 (4)	0 (0)	0.14
Cardiac cause of death—*n* (%)	2 (4)	0 (0)	0.15
All-cause death—*n* (%)	5 (10)	0 (0)	0.09

## Data Availability

The raw data collected for the purpose of this study will be made available by the authors upon request.
